# Glassy Carbon Electrode Modified with CB/TiO_2_ Layer for Sensitive Determination of Sumatriptan by Means of Voltammetry and Flow Injection Analysis

**DOI:** 10.3390/s23125397

**Published:** 2023-06-07

**Authors:** Joanna Smajdor, Beata Paczosa-Bator, Małgorzata Grabarczyk, Robert Piech

**Affiliations:** 1 Department of Analytical Chemistry and Biochemistry, Faculty of Materials Science and Ceramics, AGH University of Science and Technology, Al. Mickiewicza, 30-059 Krakow, Poland; 2 Department of Analytical Chemistry, Institute of Chemical Sciences, Faculty of Chemistry, Maria Curie-Sklodowska University, Maria Curie-Sklodowska Sq. 3, 20-031 Lublin, Poland

**Keywords:** sumatriptan, carbon black, TiO_2_, voltammetry, flow injection analysis

## Abstract

Sumatriptan is an organic chemical compound from the tryptamine group. It is used as a medicine for migraine attacks and in the treatment of cluster headaches. In this work, a new voltammetric method is proposed for highly sensitive SUM determination, using glassy carbon electrodes modified with carbon black and titanium dioxide suspension. The novelty of the presented work is the usage of the mixture of carbon black and TiO_2_ as glassy carbon electrode modifier for the first time for SUM determination. The mentioned sensor was characterized by great repeatability and sensitivity of measurements, which resulted in the obtention of a wide range of linearity and a low detection limit. The electrochemical properties of the CB-TiO_2_/GC sensor was characterized using the LSV and EIS method. The effect of different factors on the SUM peak, such as supporting electrolyte type, preconcentration time and potential, or influence of interferents, were tested using the square wave voltammetry technique. The linear voltammetric response for the analyte was obtained in the concentration range of 5 nmol L^−1^ to 150 μmol L^−1^ with a detection limit of 2.9 nmol L^−1^ for a preconcentration time of 150 s in the 0.1 mol L^−1^ phosphate buffer pH 6.0. The proposed method was successfully applied for highly sensitive sumatriptan determination in complex matrices, such as tablets, urine, and plasma, with a good recovery parameter (94–105%). The presented CB-TiO_2_/GC electrode is characterized by great stability, it was used for 6 weeks without significant changes in the SUM peak current. Amperometric and voltammetric measurements of SUM under the flow injection conditions were also performed to indicate the possibility of its fast and accurate determination with a time of single analysis of approx. 30 s.

## 1. Introduction

Migraine is a chronic condition, characterized by a unilateral localized headache, lasting approximately 4 to 72 h. It is characterized by varying degrees of severity and frequency of occurrence. The pain is aggravated by emotions or physical exertion. Photophobia, the excessive sensitivity to sounds (phonophobia) and smells (osmophobia), often accompany a migraine attack, as well as nausea and vomiting. Sometimes, before the onset of a migraine episode, the so-called aura may appear, in the form of paresthesia, visual field defects, the appearance of scotoma, or aphasia [1,2,3,4,5,6].

Triptans are a group of drugs used to treat migraine attacks. They are effective in stopping a single attack of migraine pain, but they do not prevent or cure the disease. Sumatriptan (SUM) is one of the triptan family representants. It selectively stimulates serotonin 5-HT1D receptors but does not affect other serotonin receptor subtypes. It has also been shown to reduce the activity of the trigeminal nerve. The effectiveness of triptans in the treatment of migraines has been confirmed, but because it is not fully understood how migraines occur, it is also not fully understood how they work. Sumatriptan is indicated for migraine headaches with or without aura. It eliminates or relieves migraine pain and symptoms accompanying migraine, such as nausea, vomiting, photophobia, and hypersensitivity to sounds. Its effectiveness is greater, the sooner the drug is taken, preferably immediately after the onset of a headache [7,8,9,10,11,12,13,14]. However, large doses of SUM (over 200 mg per day) may cause serious adverse effects, which include neck tension, chest pains, vertigo, seizures, sleepiness, paralysis, hypertension, limb swelling, tremor, and a rare condition called sulfhemoglobinemia [15,16,17,18]. Considering the significance of SUM usage in relieving of migraine pain and possible overdosing consequences, its quantitative determination in human body fluids is one of the interests of the analytical field. The lowest measured SUM concentration in a plasma sample was 1 ng mL^−1^ [19].

Different methods of sumatriptan determination have been reported in the literature, including spectrophotometry [20], spectrofluorimetry [21,22], or liquid chromatography coupled with various detectors [19,23,24]. Additionally, electrochemical methods, including voltammetry, are widely used for this purpose due to low detection limits, easiness of use, and low costs of analysis [21,22,23,24,25,26,27,28,29,30,31,32,33,34]. However, the electrochemical methods also have their limitations, such as higher sensitivity to the complex matrices or higher detection limits in comparison with, e.g., chromatographic methods. Additionally, some types of electrode modifiers have complicated preparation steps and require the usage of expensive solvents or nanomaterials. In the present work, a novel assay of sumatriptan determination is presented using a glassy carbon electrode (GC) modified with a carbon black and titanium dioxide layer. The electrodes modified with carbon black are well known in the field of voltammetry for the detection of pharmaceutical compounds [35,36,37]. The proposed electrode indicates very good performance for highly sensitive measurements of SUM with a wide linear range and a low detection limit. A SUM determination was performed with pharmaceutical samples, urine, and plasma with great recovery values. Additionally, sumatriptan measurements under the flow injection conditions were performed using amperometric and voltammetric detection, with good sensitivity and repeatability of registered signals, which indicated the possibility of the proposed method of automatization, which allows you to perform a large number of analyzes in a short time. The novelty of the presented work is the first time usage of a mixture of carbon black and TiO_2_ as a glassy carbon electrode modifier. Carbon black as a modifier significantly increases surface development due to its disorder structure; therefore, the analyte has more space for the preconcentration step, and the obtained signals are higher in comparison to unmodified electrodes. The addition of TiO_2_ additionally increases the SUM signal obtained on the modified electrode due to its catalytic properties. Both of the modifiers used materials that were not expensive and harmless for human health. Such modification allowed the determination of the sumatriptan with one of the best-reported limits of detection values. Additionally, the proposed procedure is also quick, and the materials and electrode preparation costs are significantly lower and less complicated, considering previous works.

## 2. Materials and Methods

### 2.1. Apparatus

Voltammetric measurements were performed using a potentiostat type M161 with an electrode stand type M164 obtained from mtm-anko (Krakow, Poland). In the measurement cell with a volume of 20 mL, an Ag/AgCl/KCl (3 mol L^−1^) electrode with a replaceable outer cover (3 mol L^−1^) was used as a reference electrode, the platinum wire was used as the auxiliary electrode, and the glassy carbon electrode (Mineral, Warsaw, Poland) modified with carbon black nanoparticles and titanium dioxide (CB-TiO_2_/GC) was used as a working electrode. Voltammograms were registered and then visualized using a dedicated EAQt software, and further analysis was performed with Origin Lab 2021b. During the preconcentration of the analyte in the voltammetric cell, stirring was performed using the Teflon coated magnetic bar with an approx. speed of 500 rpm. pH measurements were performed using the Elmetron CX-705 multi-function meter (Elmetron, Zabrze, Poland). The sonication of the prepared solutions was carried out using an ultrasonic bath (Intersonic, IS-1K, Olsztyn, Poland).

The flow system consisted of a 0.05 L solution reservoir, 800 Dosino pump with 900 Touch control panel (both Metrohm, Herisau, Switzerland) and a Rheodyne Model 7010 sample injection valve with a sample loop of 100 μL (both IDEX Health & Science, Middleboro, MA, USA) and a flow detector. All interconnections were made of 0.5 mm Tygon tubing. The length of tubing between the valve and the detector was 1 m. The wall—jet detector was used in the form of screen-printed carbon electrodes with built in reference electrodes (Metrohm, Herisau, Switzerland).

The electrical impedance spectroscopy measurements (EIS) were conducted with the use of an Autolab General Purpose Electrochemical System (Eco Chemie AUT32N.FRA2-AUTOLAB, ΩMetrohm, Switzerland). Studied GC electrodes modified with different modifiers were connected for measurements as working electrodes into a three-electrode cell with an Ag/AgCl/KCl (3 mol L^−1^) electrode as the reference electrode and a glassy carbon rod as the auxiliary one. All data analysis was carried out and interpreted using NOVA software. Measurements were performed in 1 mmol L^−1^ K_3_[Fe(CN)_6_] in 1 mol L^−1^ KCl using the sinusoidal signals of frequency ranging from 100 kHz to 10 mHz and 50 mV amplitude, superimposed on the formal potential of K_3_[Fe(CN)_6_].

### 2.2. Chemicals and Glassware

All chemical reagents were purchased and used without any additional purification. A standard stock solution of 0.01 mol L^−1^ sumatriptan (≥98%, Sigma Aldrich, St. Louis, MO, USA) was prepared dissolution in double deionized water and stored in the fridge between measurements. Solutions with lower SUM concentration were prepared daily. Phosphate buffer (1 mol L^−1^, pH 6.0) was prepared by mixing the proper amounts of KH_2_PO_4_ and K_2_HPO_4_ (both Merck, Darmstadt, Germany). Carbon black nanoparticles (CB) characterized by the following parameters, surface area: 100 m^2^g^−1^ and average particles size: 30 nm, were purchased from 3D-nano (Poland), and dimethyloformamide (DMF) was obtained from Sigma Aldrich (St. Louis, MO, USA). Freeze dried urine (Medidrug Basis-line U) was purchased from Medichem (Steinenbronn, Germany), and human plasma was purchased from Biowest (Nuaillé, France). Double-deionized water was used to prepare all solutions.

### 2.3. Sample Preparation

#### 2.3.1. Tablet

Sumatriptan concentration was measured in pharmaceutical formulation Frimig (Orion, 50 mg sumatriptan per tablet) purchased in the local drugstore. For the quantitative analysis, three of them were crushed in the mortar and then dissolved in a volumetric flask with double-distilled water. For the quantitative analysis, a Frimig solution was filtered through syringe filters with a regenerated cellulose (RC) membrane and a pore size of 0.45 μm (Biosens, Warsaw, Poland), and solutions with lower concentration were prepared.

#### 2.3.2. Urine

For highly sensitive SUM measurements in the urine, a commercial freeze-dried urine reagent was purchased from Medidrug (Barcelona, Spain) and prepared according to the manufacturer’s procedure. The content of the vial was completely dissolved with double-distilled water and filtered through an RC syringe filter with a pore size of 0.45 μm (Biosens). After preparation, a sample was used for the measurement of SUM.

#### 2.3.3. Plasma

For highly sensitive SUM measurements in the plasma, a commercial plasma sample was purchased from Biowest (France) and stored in the freezer at −20 °C. In order to remove potentially interfering protein components from the sample, 800 μL of plasma was mixed with 200 μL of 10% trichloroacetic acid (TCA), shaken for 2 min on the vortex (Biosan) and then centrifuged for 30 min with an approximate speed of 10,000 rpm (Eppendorf, Hamburg, Germany). The obtained supernatant was filtered through syringe filters with the RC membrane and a pore size of 0.45 μm (Biosens) and used in further measurements.

### 2.4. Modifier Suspension Preparation

The modifier suspension was obtained by mixing the carbon black (3-D nano) with the TiO_2_ powder with a weight proportion of 1:0.5. The loose components were crushed in the mortar in order to obtain full homogenization and transferred to the Eppendorf tube. Dimethylformamide (DMF) (Sigma) was used as the solvent in such modifier mixtures. The prepared suspension was shaken in the ultrasonic bath for 45 min before being placed on the electrode surface in order to achieve a fully homogeneous solution.

### 2.5. Working Electrode Preparation

All measurements were performed on a GC electrode with a diameter size of 3 mm (Mineral, Poland) modified with CB-TiO_2_ suspension. Prior to the modification, the GC electrode surface was polished on the polishing pad with 0.3 μm alumina slurry (Buechler, Chicago, IL, USA) and then carefully rinsed with the double-distilled water in order to remove the leftovers of the alumina Al_2_O_3_ powder. Then, the GC electrode was transferred to the beaker filled with methanol and placed in the ultrasonic bath for 3 min. After complete evaporation of the methanol leftovers, 10 μL of CB-TiO_2_ suspension was dropped, using the automatic pipette (Eppendorf), on the GC electrode surface and left to evaporate the solvent for approx. 3 h. After such preparation, the electrode was ready for SUM quantitative measurements.

### 2.6. Measurement Procedure

Sumatriptan quantitative analyses were performed using the square wave voltammetry (SWV) technique. Measurements were conducted in a 0.1 mol L^−1^ phosphate buffer pH 6.0 as a supporting electrolyte. The instrumental parameters of the highly sensitive SUM measurements were as follows: sampling and waiting time t_p_ = t_w_ = 5 ms, step potential E_s_ = 5 mV, pulse amplitude dE = 50 mV, and frequency f = 50 Hz. The voltammograms were registered in the potential range from 100 to 1100 mV, after the preconcentration step described by the parameters: E_acc_ = 100 mV and t_acc_ = 10 s. Between each voltammogram registration, a rest period of 20 s was strictly required in order to obtain the high repeatability of the SUM signals.

## 3. Results and Discussion

### 3.1. CB/TiO_2_ Modified GC Electrode Electrochemical Characterization

**Figure 1:**
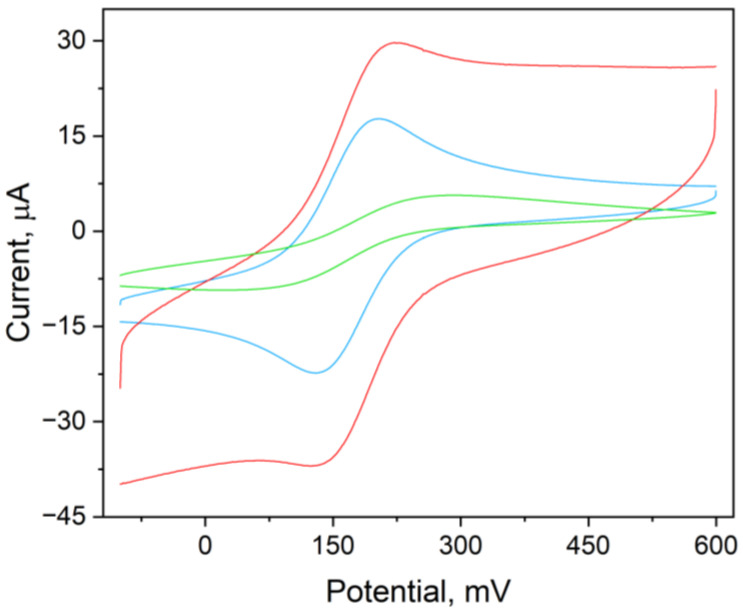
Cyclic voltammograms of 1 mmol L^−1^ K_3_[Fe(CN)_6_] in 1 mol L^−1^ KCl for CB/GC electrode (blue line), TiO_2_/GC electrode (green line) and for CB/TiO_2_/GC electrode (red line) with a scan rate of 100 mV s^−1^.

The electrochemical behavior of the CB/TiO_2_ modifier layer on the GC electrode was investigated. For this purpose, cyclic voltammograms of the glassy carbon electrode modified with only carbon black, only titanium dioxide, and with both components were registered in 1 mmol L^−1^ K_3_[Fe(CN)_6_] in 1 mol L^−1^ KCl. The voltammograms registered for each electrode with a scan rate of 100 mV s^−1^ are presented in Figure 1.

For the electrode modified with CB and CB/TiO_2_, a couple of well-defined redox peaks were observed. For the TiO_2_ layer, the obtained peaks were stretched out, and the determination of the values of the anodic and cathodic peak potential and the current was not obvious. The distance between the cathodic and anodic peak (∆E_p_) was 73 mV for the CB/GC electrode and 226 mV for TiO_2_/GC electrode. For the GC modified with both components, the value of the difference was intermediate and amounted to 95 mV. Moreover, the peak current for the CB/TiO_2_/GC electrode was definitely larger in comparison to separate modifiers applied to the GC surface. In Table 1, a comparison of anodic and cathodic peak separation and current for modified and unmodified electrodes is presented. The area (*A*) of the modified GC electrode active surface was also calculated using a transformed Randles—Sevcik equation:

A=Ipkn32D12v12C

where:

*k*—constant value of 269,000;

*I_p_*—peak current;

*n*—number of transferred electrons;

*D*—diffusion coefficient (7.6∙10^−6^ cm^2^ s^−1^);

*v*—scan rate;

*C*—concentration of depolarizer.

**Figure 2:**
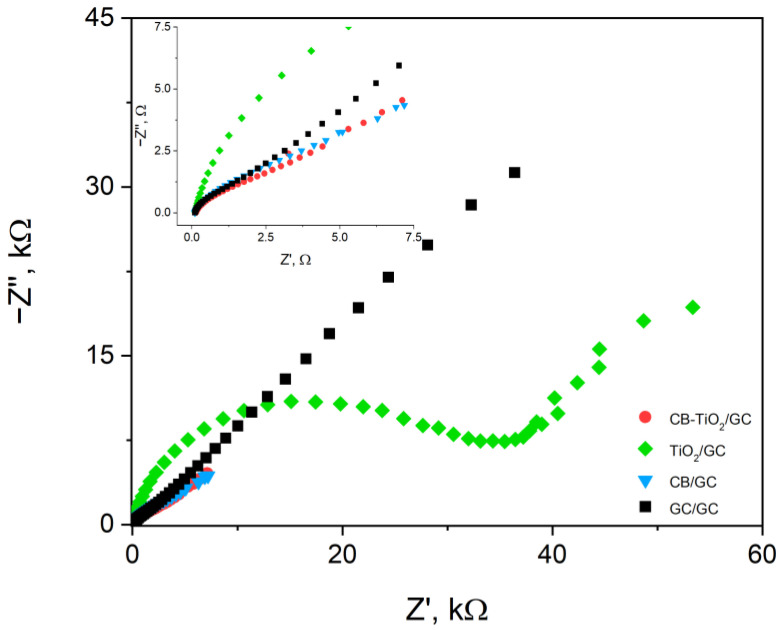
Nyquist plot obtained in 1 mmol L^−1^ K_3_[Fe(CN)_6_] in 1 mol L^−1^ KCl for GC electrode (black) CB/GC electrode (blue), TiO_2_/GC electrode (green), and for CB/TiO_2_/GC electrode (red).

As seen in Table 1, the addition of TiO_2_ to the carbon black increases the working area of the modified electrode, thus it is improving its sensitivity. The morphology of the CB, TiO_2_, and CB/TiO_2_ layer obtained using SEM microscopy is also presented in Figure 2.

Performing the EIS measurement allowed the obtention of the capacitance (*C_eff_*) and resistance (*R_ct_*) parameter for modified and unmodified GC electrodes. The capacity was calculated using data from the NOVA fitting tool and the equation:

Ceff=Y01N·1Rs+1RpN−1N

The resistance parameter calculated using the values of the *C_eff_* and *R_ct_* parameter are presented in Table 2. The results of EIS measurements are presented in Figure 2.

Considering the results obtained from LSC and EIS, it is visible that the highest capacitance along with the lowest charge transfer resistance is measured for CB/TiO_2_/GC, and it is possible to say that it is the outcome of the combination of black carbon particles with titanium dioxide. Both electrochemical techniques (LSV and EIS) allowed for the observation of more favorable properties of GC electrodes modified with a mixture of CB/TiO_2_ over electrodes modified with separate compounds and non-modified GC electrodes, which results in better analytical performance.

### 3.2. Voltammetric Behaviour of Sumatriptan on CB-TiO_2_/GC Electrode

**Figure 3:**
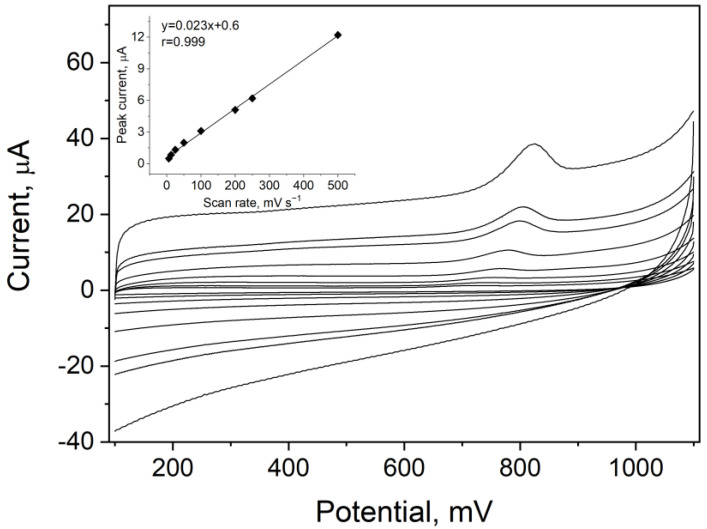
Cyclic voltammograms of 1 μmol L^−1^ sumatriptan measured in a 0.1 mol L^−1^ phosphate buffer (pH 6.0) on the CB-TiO_2_/GC electrode. The scan rate values were as follows: 6.3; 12.5; 25; 50; 100; 200; 250; and 500 mV s^−1^.

The cyclic voltammetric studies for 1 μmol L^−1^ SUM behavior on the CB-TiO_2_/GC electrode were performed in the range of the scan rate value between 6.3 and 500 mV s^−1^ in a 0.1 mol L^−1^ phosphate buffer pH 6.0 and a potential window from 100 to 1100 mV. The results of the measurements are presented in Figure 3. During the measurement, only the anodic SUM peak was registered, which implies that the described oxidation process is irreversible. The linear relationship between SUM peak current and the scan rate value was obtained, which suggests that the oxidation process on the electrode surface is controlled by the adsorption. Considering the LSV SUM voltammograms, we tried to elucidate the course of the oxidation reaction. The number of electrons exchanged during the oxidation process was calculated using the slope value from the dependence of peak current and natural logarithm of the scan rate. Considering α value as 0.5, the number of transferred electrons could be calculated as two. To confirm the result obtained, the number of electrons was also calculated using the following equation:

αn=0.048Ep−Ep12

The obtained nα value was equal to 0.99, which, considering the α value as 0.5, gives the result of two electrons exchanged in the SUM oxidation reaction. Therefore, the previous calculation has been confirmed.

**Figure 4:**
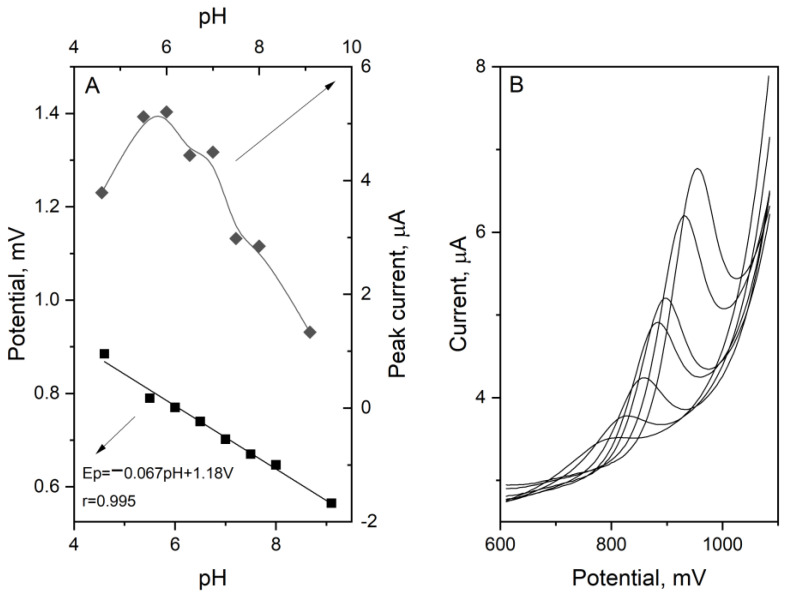
Plots of the sumatriptan peak current and potential dependence on the supporting electrolyte pH in the range 4.6–9.1 (**A**) and corresponding SWV voltammograms of 0.5 μmol L^−1^ sumatriptan for pH in the range 5.5–8.0 measured in a 0.1 mol L^−1^ phosphate buffer (**B**). Instrumental parameters as in point 2.6.

To fully determine the course of the reaction, the SUM peak behavior in different electrolyte pH was examined. In total, 0.1 mol L^−1^ of phosphate buffer was examined in the pH range from 4.5 to 9.1. In Figure 4, the dependence between SUM peak current and potential on the pH value is presented with corresponding voltammograms. The anodic SUM peak current was shifting towards higher potential values with the decrease in the pH value, which can be described using the following equation:

$$E_{p}=-0.067\;pH+1.15\;V$$

**Scheme 1:**
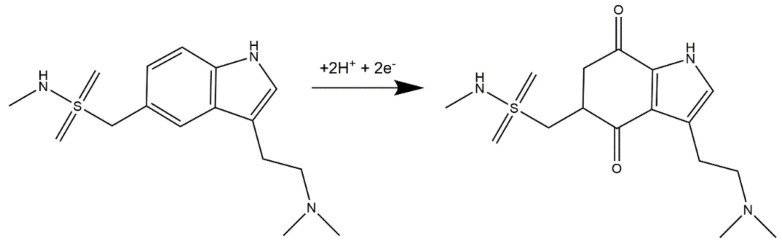
Possible scheme of sumatriptan oxidation of the glassy carbon electrode modified with carbon black and titanium dioxide suspension.

The obtained slope value is close to the theoretical 0.059, which implies that an equal number of protons and electrons are involved in the SUM oxidation reaction on the surface of CB-TiO_2_/GC electrode. The scheme of the possible oxidation mechanism is presented in Scheme 1.

**Figure 5:**
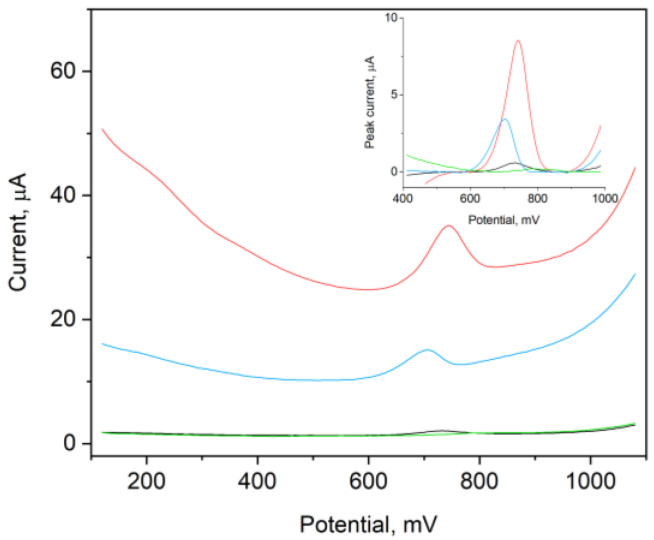
Comparison of voltammograms obtained for the anodic peak of 1 μmol L^−1^ sumatriptan, measured in the 0.1 mol L^−1^ phosphate buffer (pH 6.0) on the surface of glassy carbon electrode (black line), glassy carbon electrode modified with carbon black (blue line), glassy carbon electrode modified with titanium dioxide (green line), and glassy carbon electrode modified with carbon black/titanium dioxide (red line) before and after background correction. Instrumental parameters as in point 2.6.

In order to provide the highest sensitivity and repeatability of SUM signals, the modification of GC electrode was used in the form of CB and TiO_2_ suspension in the DMF as described in points 2.4 and 2.5. The effect of the modifier volume on the GC electrode on the SUM peak was investigated in this respect. In Figure 5, SUM signals obtained for GC, CB/GC, TiO_2_/GC, and CB-TiO_2_/GC electrode are presented. The highest signals with satisfying repeatability have been obtained using a 10 μL volume of CB-TiO_2_ modification layer, therefore this modifier was used in further measurements.

### 3.3. Influence of the Preconcentration Time and Potential on Sumatriptan Peak

In order to achieve the best analytical performance of the presented sensor for SUM high sensitive determination, the optimal value of preconcentration time and potential was selected on the basis of performed measurements. The preconcentration potential was tested in the range of −200 to 500 mV for a preconcentration time of 20 s. A time of 20 s was chosen due to visible differences between SUM peak current for its different concentration. SUM peak did not exhibit a strong dependence on this parameter, therefore the preconcentration potential was chosen as 0 mV for further measurements.

**Figure 6:**
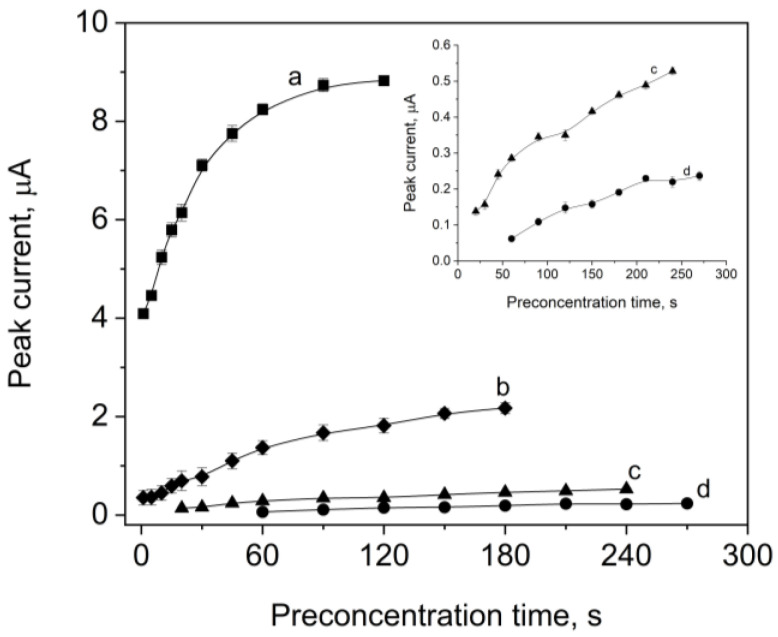
Dependence of the sumatriptan peak current on the value of preconcentration time. SUM concentrations as follows: (a) 0.5 μmol L^−1^; (b) 0.05 μmol L^−1^; (c) 0.01 μmol L^−1^, and (d) 5 nmol L^−1^. Other instrumental parameters are the same as in point 2.6.

The dependence of preconcentration time of the SUM peak is presented in Figure 4. Measurements were performed on the GC electrode modified with CB and TiO_2_ for six SUM concentrations. As can be seen in Figure 6, in all considered cases, the SUM peak current rose with the rise in preconcentration time, until reaching the maximum value. After that, the increase in the current value was inhibited and stayed at a stable level.

### 3.4. Influence of the Supporting Electrolyte Composition on the Sumatriptan Peak

In order to choose the most suitable environment for high sensitive SUM determination, the following electrolytes were tested: 0.1 mol L^−1^ phosphate buffers with pH values of 6.0 and 7.5; 0.5 mol L^−1^ acetate buffers with pH values of 3.8, 4.5, and 5.5; 0.1 mol L^−1^ sodium tetraborate solution with a pH of 10; 0.5 mol L^−1^ ammonia buffer with a pH of 8.2; 0.1 mol L^−1^ perchloric acid and 0.1 mol L^−1^ sodium hydroxide. Comparison of the obtained voltammograms led to the conclusion that the best properties of the SUM peak are exhibited in the 0.1 mol L^−1^ phosphate buffer with a pH of 6.0, therefore this supporting electrolyte has been chosen for further measurements.

### 3.5. Interferences

Different organic and non-organic interferents influence on SUM peak were investigated during the measurements under optimized conditions. The results showed that the additions of the following metals Zn (II), Cd (II), Al (III), Pb (II), Fe (III) (2 μmol L^−1^), Mg (II), Ca (II) and the addition of non organic anions and cations, such as SO_4_^2+^, NO_3_^−^ (1 mmol L^−1^), and CO_3_^2−^ (0.2 mmol L^−1^), did not cause a difference in the SUM peak current and potential value. However, a difference in the SUM signal was observed for the addition of 2 mol L^−1^ of Mn (II) (decrease of 23%) and of 1 mmol L^−1^ of NH_4_^−^ and Cl^−^ (13% increase and 9% decrease, respectively). The serious decreasing influence of Mn (II) is caused by its oxidation on the surface of the CB/TiO_2_/GC electrode. The Mn (II) peak of its oxidation from the form (II) to (IV) is placed at the potential of about 750 mV, which makes it difficult for SUM signal interpretation, which is placed near the potential of 800 mV. Therefore, after background correction, the result of Mn (II) ions is visible in the decrease in SUM peak current. Moreover, the addition of organic compounds that may possibly occur in pharmaceutical samples or body fluids was tested. The addition of glucose, saccharose (0.1 mmol L^−1^), lactose monohydrate (60 μmol L^−1^), ascorbic acid, citric acid (0.2 mmol L^−1^), aspartame, microcrystalline cellulose, and magnesium stearate (20 μmol L^−1^) did not interfere with the SUM peak. Only caffeine addition in the concentration of 20 μmol L^−1^ caused a 9% decrease in SUM peak.

### 3.6. Analytical Performance

**Figure 7:**
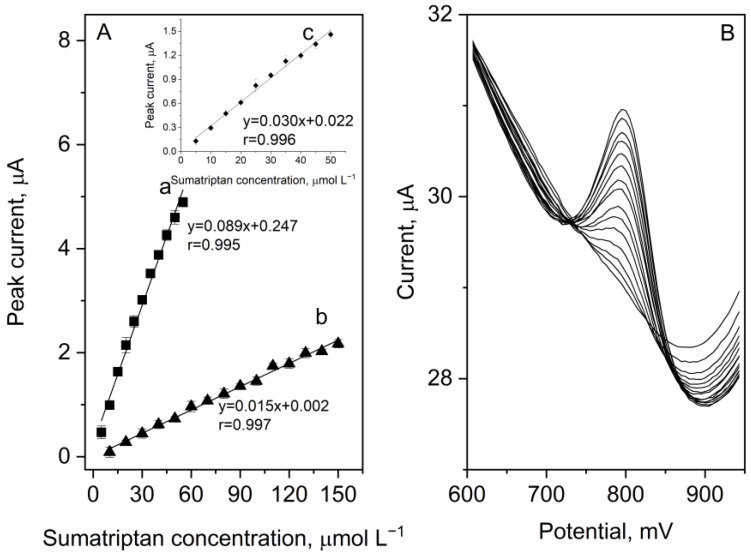
SWV sumatriptan calibration curves registered for the preconcentration times (a) 10 s; (b) 45 s; (c) 150 s in 0.1 mol L^−1^ phosphate buffer (pH 6.0) (**A**) and corresponding voltammograms obtained for preconcentration time 45 s in the concentration range of 10–150 nmol L^−1^ (**B**). Other instrumental parameters are the same as in point 2.6.

The square wave voltammetry (SWV) technique was used for high sensitivity of SUM determination in the nanomolar concentration. The SWV SUM voltammograms in the concentration range of 5 nmol L^−1^ to 150 μmol L^−1^ and corresponding calibration curves are presented in Figure 7. The linearity of the SUM peak of the CB-TiO_2_/GC electrode was very good in the whole examined range. The detection limit obtained for a short preconcentration time of 10 s was 35 nmol L^−1^ with a linearity of up to 55 μmol L^−1^. The lowest detection limit was obtained for 150 s preconcentration time, and it was equal to 2.9 nmol L^−1^ (slope of the regression line 0.03 [μA (10^−9^ mol L^−1^)^−1^], intercept 0.02 μA), which is a very good result in comparison with other electrochemical methods (Table 3). The reproducibility of the method was measured and expressed as an RSD parameter for the SUM concentration of 0.1 mol L^−1^. RSD was equal to 3.4%, which is a very good result for the modified electrode.

To validate the obtained results, the proposed method was applied for high sensitive SUM determination in complex matrices, such as pharmaceutical formulation, urine, and plasma. The samples were prepared according to the procedure in point 2.3 and added to the supporting electrolyte with a final dilution value of 20 (for urine samples) and 30 (for plasma samples). Quantification of SUM was performed by the standard addition method and the results with the recovery value are presented in Table 4. Measured recovery was good, specified in the range of 94 to 105%. Therefore, the analytical usefulness of the proposed voltammetric method using carbon black—titanium dioxide modified GC electrode was confirmed for SUM determination in different samples.

Additionally, studies of SUM biodegradability were performed. Three tablets of SUM were crushed and placed in the river sample from Rudawa river, collected in the center of Krakow. Water was filtered on the hard quantitative filter in order to get rid of solid particles. The suspension was placed in the daylight with access to the air for four weeks. The SUM content was examined using proposed voltammetric assay the day the experiment started (day 0) and then after 7, 14, 21, and 28 days. The results of the analysis showed that the SUM content in the suspension was constant, therefore it is possible to say, that SUM does not undergo a process of degradation in the natural water sample with access to oxygen and UV radiation.

### 3.7. Flow Injection Analysis with Amperometric and Voltammetric Detection

**Figure 8:**
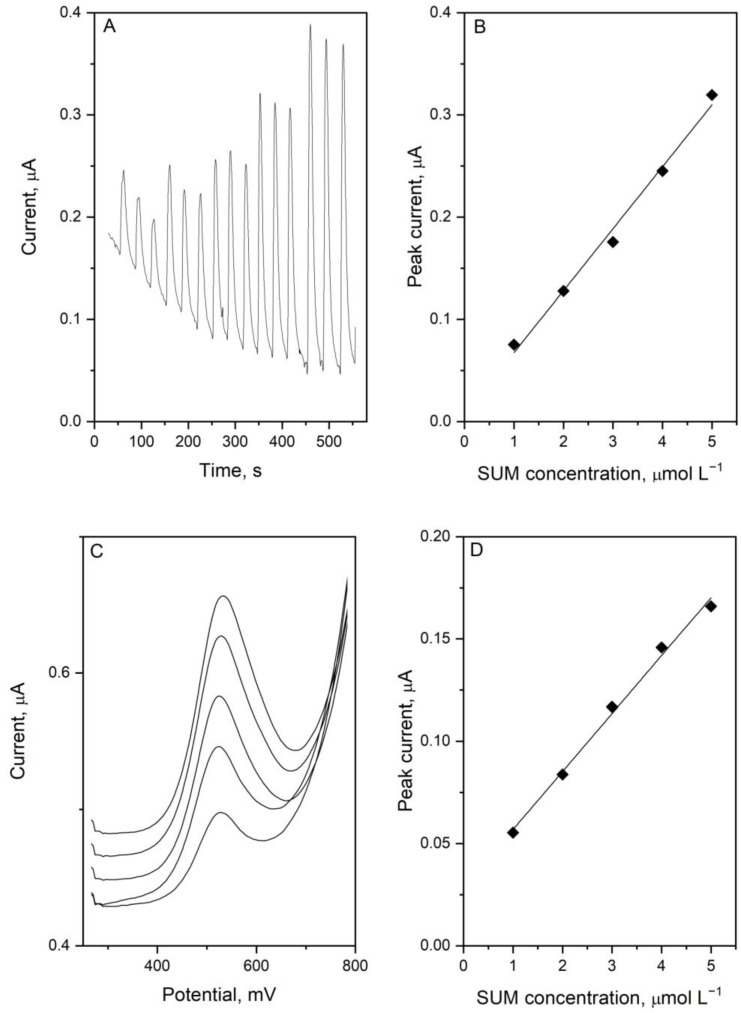
Sumatriptan calibration plots in the concentration range from 1 to 5 μmol L^−1^ on modified SPCE in the amperometric parameters of measurements (**A**,**B**) and in the voltammetric parameters of measurements (**C**,**D**). All experiments were performed under flow injection conditions.

An amperometric determination of SUM under flow injection conditions was also performed. The characteristic parameters of FIA analysis, such as the working potential and flow rate, were investigated in a wide range of values. The optimal potential was chosen as 900 mV, and the calibrations were registered with an optimal flow rate equal to 1.5 mL min^−1^. Additionally, the voltammetric conditions of SUM determination on the modified SPCE were used in the experiment. The SUM signal was recorded on the modified electrode surface 20 s after the injection of the sample into the system, providing that the whole volume of the sample reached the sensor. The measurements were performed on a SPCE (screen-printed carbon electrode) modified with a CB-TiO_2_ suspension. The linear response of the SUM under the flow conditions was registered for concentrations ranging from 1 to 5 μmol L^−1^. In Figure 6, the diagram of SUM calibration is presented with the calibration plot both for amperometric (A, B) and voltammetric (C, D) detection on the SPCE electrode. The RSD of performed amperometric measurements was equal to 4.4%, and for voltammetric detection, it was equal to 2.6%. According to the available literature, CB-TiO_2_-modified SPCE was used for the first time for SUM determination in flow analysis conditions. The usage of the proposed procedure allowed to significantly reduce the time of a single analysis while maintaining a high sensitivity of measurements, which led to the conclusion that the proposed assay may be useful for quality control of SUM content in samples. The time of single sample analysis for both amperometric and voltammetric detection was about 30 s, and the whole calibration procedures presented in Figure 8 took about 10 min.

## 4. Conclusions

The new SWV method for the highly sensitive SUM determination is proposed using a GC electrode modified with carbon black and titanium dioxide. The novelty of the presented work is the usage of the mixture of carbon black and TiO_2_ as a glassy carbon electrode modifier for the first time. The proposed sensor, consisting of two components, was characterized by more favorable properties than when the components were used separately, which was proven through the LSV and EIS measurements. Such modification, due to its physical properties, allows the determination of the sumatriptan with one of the best-reported limits of detection values. The lowest obtained detection limit for 150 s of SUM preconcentration was equal to 2.9 nmol L^−1^. The procedure described was successfully applied for SUM determination in complex matrices such as tablets, urine, or plasma with very good recovery parameters (94–105%). Moreover, SUM determination was also successfully performed on the SPC electrodes during the flow measurements. The presented CB-TiO_2_/GC electrode exhibited excellent stability, as used for 6 weeks without significant change in the SUM peak current (for the SUM concentration of 0.5 μmol L^−1^, the decrease in the signal was of about 7.3%, and there was no change in the peak potential value). The experiments performed confirmed the possibility of measuring SUM under flow injection conditions, which allows to significantly reduce the time and cost of analysis. Moreover, the flow injection measurements allow us to analyze a great deal of samples in a relatively short time. Additionally, the material and electrode preparation costs are significantly lower. Considering all obtained results, it is possible to say that the described voltammetric and amperometric methods are suitable for fast, simple, and accurate determination of SUM using GC electrodes modified with a carbon black and titanium dioxide layer. The proposed sensor and methodology are an excellent alternative to the measurements described so far in the literature.

## Figures and Tables

**Table 1:** The electrochemical parameters obtained for the GC electrode modified with carbon black, titanium dioxide, and carbon black/titanium dioxide layer with a scan rate of 100 mV s^−1^.

	E_pa_, mV	E_pc_, mV	∆E_p_	I_pa_, µA	I_pc_, µA	I_pa_/I_pc_	A, cm^3^
CB	204	131	73	19.76	21.46	0.92	0.084
TiO_2_	271	45	226	5.14	8.48	0.61	0.022
CB/TiO_2_	224	129	95	21.58	23.85	0.90	0.092

**Table 2:** The capacitance and charge transfer resistance parameters values obtained for both the modified and unmodified electrode.

	GC	TiO_2_/GC	CB/GC	CB-TiO_2_/GC
C, µF	0.692	0.057	0.307	42.3
R_ct_, kΩ	1.60	31.9	2.50	1.42

**Table 3:** Comparison of sumatriptan determination methods.

Electrode	Technique	Sensitivity	Linear Range, µM	LOD, mol L^−1^	Reference
Pt-ZONPs/CPE	CV	-	0.010–55	3·10^−9^	[25]
PAP/GCE	SWV	-	1.0–100.0	0.294·10^−6^	[26]
Gr/AuNP/NAF/GCE	AdSDPV	0.48 µA/µM	0.002–41.20	7.03·10^−10^	[27]
CuO/SPE	DPV	-	0.33–3.54	0.066·10^−6^	[28]
ZnO/NiO/Fe_3_O_4_/MWCNTs/GCE	SWV	-	0.006–380.00	2·10^−9^	[29]
MXene/MWCNT/chitosan/GCE	AdSDPV	-	0.0033–61	0.00042·10^−6^	[30]
CuNPs/poly-melamine/GCE	DPV	1.14 µA/µM	0.08–0.58	0.025·10^−6^	[31]
Ni–Co LDH/SPE	DPV	0.1017 µA/µM	0.01–435.0	0.002·10^−6^	[32]
MIP/Sol-Gel/PWA/rGO/PGE	AdDPV	10.97 µA/µM	0.02–3	4·10^−9^	[33]
Fe_3_O_4_@ZIF-8/SPGE	DPV	0.1013 µA/µM	0.035–475.0	0.03·10^−6^	[34]
CB-TiO_2_/GC	SWV	0.03 µA/nM	0.005–150	2.9·10^−9^	This work

Pt-ZONPs/CPE—ZrO_2_ nanoparticles modified carbon paste electrode; PAP/GCE—Glassy carbon electrode modified with p-aminophenol; Gr/AuNP/NAF/GCE—Graphene-gold nanoparticles-Nafion modified glassy carbon electrode; CuO/SPE—Copper oxide based disposable screen-printed sensor; ZnO/NiO/Fe_3_O_4_/MWCNTs/GCE—Glassy carbon electrode modified with the multiwalled carbon nanotubes decorated with ZnO, NiO and Fe_3_O_4_ nanoparticles; MXene/MWCNT/chitosan/GCE—Multi-walled carbon nanotubes supported on Ti_3_C_2_–MXene and chitosan composite film–based glassy carbon electrode; CuNPs/poly-melamine/GCE—Polymeric surface decorated with Cu nanoparticles; Ni–Co LDH/SPE—Ni-Co layered double hydroxide hollow nanostructures modified screen printed electrode; MIP/Sol-Gel/PWA/rGO/PGE—Molecular imprinted polymer/sol-gel/polyoxometalate/rGO modified pencil graphite electrode; Fe_3_O_4_@ZIF-8/SPGE—Magnetite nanoparticles/Zeolitic imidazolate frameworks modified screen-printed electrode; CB-TiO_2_/GC—Carbon black—titanium dioxide modified glassy carbon electrode.

**Table 4:** Results of sumatriptan determination in different samples.

Sample	Added, μmol L^−1^	Found ±, μmol L^−1^	Recovery, %
Tablet	0	0.09 ± 0.01	-
	0.1	0.20 ± 0.03	103
	0.2	0.29 ± 0.03	102
	0.3	0.39 ± 0.04	98
Urine (20 × diluted)	0	ND	-
	1.0	0.95 ± 0.05	95
	2.0	2.09 ± 0.05	105
	3.0	2.99 ± 0.02	99
	4.0	3.96 ± 0.04	99
Plasma (30 × diluted)	0	ND	-
	0.5	0.47 ± 0.06	94
	1.0	1.05 ± 0.01	105
	1.5	1.56 ± 0.03	104
	2.0	1.94 ± 0.02	97

## Data Availability

The data presented in this study are available on request from the corresponding author.
